# Investigating the Deinking of Polyethylene Post-Consumer Waste Flakes Using Industrially Applicable Surfactants

**DOI:** 10.3390/polym18172172

**Published:** 2026-09-05

**Authors:** Steven Zimmer, Lisa Leuchtenberger-Engel, Achim Grefenstein, Rainer Dahlmann

**Affiliations:** Institute for Plastics Processing (IKV) in Industry and Craft, RWTH Aachen University, Seffenter Weg 201, 52074 Aachen, Germany; steven.zimmer@ikv.rwth-aachen.de (S.Z.); lisa.leuchtenberger@ikv.rwth-aachen.de (L.L.-E.);

**Keywords:** polymer recycling, circular economy, waste management, deinking, statistical analysis

## Abstract

In recycling of plastic packaging, the deinking of post-consumer waste (PCW) is a key technology to achieve high-quality recyclates that can be used in demanding applications. Removing printing inks minimises the contamination in the mechanical recycling process, enabling the production of (semi)-transparent recycled films with low odour and fewer defects. While the deinking of uniformly printed model films is thoroughly researched and the general mechanism well understood, these findings are not readily transferable to industrial implementation on household PCW. This study systematically investigates the deinking of household PCW flakes using industrially available surfactants. Deinking parameters (water temperature, washing time, lye concentration) are varied with no surfactant added as well as for four industrially available surfactant formulations. The deinking efficiency is determined via an image-based statistical analysis. Via a multiple linear regression, median grey values of the experiments allow insights into the deinking mechanism of PCW flakes. Depending on the surfactant and deinking parameters, either the hydrolysis of the binding agent by sodium hydroxide (NaOH) or the surfactant-based decrease of interfacial energy on the printed surface proved dominant for the overall deinking mechanism.

## 1. Introduction

Among the packaging solutions produced in and imported into the EU28+2, flexible polyolefin packaging has the highest capacity with around 11 Mt in 2020, with 9 Mt amounting to PE only [1]. The vast range of options for introducing functional layers (e.g., barriers against moisture, light, and oxygen), excellent printability, and low cost render flexible PE the preferred choice for many packaging applications [2,3,4,5]. Given that the lifespan of flexible packaging is typically limited to a few weeks, it constitutes the predominant PCW flow in terms of quantity [6,7]. However, functional layers cause great inhomogeneity of this waste flow, including foreign polymers such as polypropylene (PP), polyethylene terephthalate (PET), and ethylene vinyl alcohol (EVOH), as well as metal barriers and printing inks [7,8,9]. These foreign materials, together with non-intentionally added substances (NIAS), cause a plethora of undesired side effects and reactions during mechanical recycling, limiting the recycling materials to applications of low requirement [10].

The packaging and packaging waste regulation (PPWR) restricts the use of many foreign materials and requires the increased implementation of mono-material solutions [11,12,13,14]. While the PPWR will expectedly minimise the challenges posed by some foreign materials, no regulations for printing inks are included. These generally make up 2 to 4 wt.% of flexible packaging and serve marketing purposes as well as the mandatory product and consumer information [15,16]. Current printing inks predominantly contain nitrocellulose (NC) as a binding agent, which is known to degrade at temperatures above 160 °C, yielding volatile organic compounds (VOC) as well as substances that are dark in colour and, more importantly, harmful to health [17,18,19,20].

**Figure 1:**
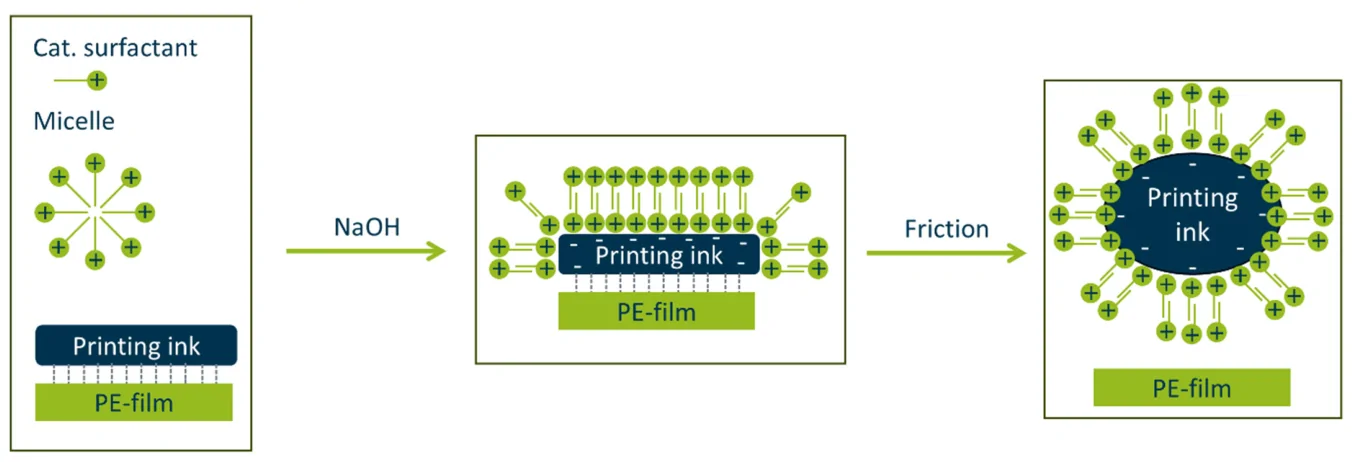
Deinking mechanism for physically drying inks, drawn after [24]. NaOH interacts with the binding agent in the ink layer, creating negative charges on the ink layer surface. The hydrophilic part of cationic surfactants adsorbs to the surface due to Coulomb forces, weakening hydrogen bonds as well as the van der Waals forces. The now swollen ink layer loses adhesion to the plastic film and gets solubilised into the aqueous medium, where it is stabilised in micelles [24,27].

Consequently, the complete removal of printing inks prior to regranulation offers the potential to produce recyclates of superior quality in terms of mechanical and optical properties as well as odour [21,22,23]. The deinking of film flakes has attracted considerable attention in recent years, with multiple research groups investigating washing parameters and unravelling the mechanisms of ink removal [5,23,24,25,26]. In 2007, Chotipong et al. proposed the deinking mechanism using a cationic surfactant in alkaline media, which was further described in 2023 by Guo et al., as shown in Figure 1 [24,27].

Since 2024, the DIN SPEC 91496 has proposed a methodological procedure for deinking experiments on the laboratory scale. Deinking efficiency heavily depends on washing parameters such as friction, pH, surfactant formulation, and water temperature [5,22,24]. Likewise, film properties as the type of binding agent and general printing ink formulation as well as the occurrence of functional layers (e.g., metal barriers, laminations) can influence deinking [28].

In consideration of the PPWR’s requested minimum amounts of post-consumer recyclate (PCR) use for plastic packaging, ranging from 10% for contact-sensitive packaging to 35% for other single-use packaging products, the investigation of deinking on PCW flakes is highly sought after in both scientific and industrial fields. However, the deinking of printed films has so far only been systematically investigated and evaluated using surface-printed mono-layer model films. This experiment design allows for quick and simple evaluation of ink removal but compromises on the applicability to real-life PCW flakes, namely the influence of contaminants from waste residues on the deinking and the non-transferable evaluation process [2,29]. The International Commission on Illumination Lab (CIELab) method, used to calculate the deinking efficiency, utilises an unprinted film sample as reference. This is hardly applicable to inhomogeneous (i.e., printed, transparent, laminated, mass-coloured) PCW flakes that come in the form of rolled-up, torn, and folded flakes [5,24,29].

Recently, our group presented a methodology for the straight-forward quantification of ink removal that allows the comparison of deinking experiments on PCW flakes while still allowing a lab-scale approach [29]. This was achieved by scanning small amounts of flakes with transmitted light and converting the images to the 8-bit grey scale. Higher grey values are an indicator for higher flake transparency. Consequently, grey value distributions can be statistically evaluated and correlated to the efficiency of a deinking experiment. The method proved reproducible and robust despite the flakes’ inhomogeneous structure and film design. Within the parameters investigated, there was a significant shift towards higher grey values (i.e., higher flake transparency) for higher water temperatures (30 °C < 60 °C < 80 °C) and longer washing times (15 min < 60 min < 120 min). This study investigates whether the presented evaluation method contributes to the transfer of comprehensive deinking insights to industrial deinking applications on PCW flakes. The deinking of PCW flakes is systematically investigated using cetrimonium chloride (CTAC), which is the proposed surfactant in the DIN SPEC 91496, as well as additional cationic and non-ionic surfactants that are industrially available. Furthermore, lye concentration and washing parameters are varied with the aim of identifying beneficial parameters for the deinking of PCW flakes. The full factorial design is statistically analysed by multiple linear regression, allowing a deeper understanding of the deinking mechanism on PCW flakes. These findings may serve as a basis for adapting industrial washing processes, thereby enabling the production of recycled materials with improved qualities through effective deinking.

## 2. Materials and Methods

This chapter includes the utilised materials, the pre-treatment of the PCW flakes, the deinking procedure, and the evaluation of the ink removal.

### 2.1. Materials and Pre-Treatment of PCW Flakes

PCW flakes from the “DSD310” fraction were kindly provided by Der Grüne Punkt GmbH & Co. KG, Cologne, Germany. In this fraction, household waste is sorted for flexible PE films. The films are shredded to a size of 40 to 60 mm and separated from coarse impurities and labels using a metal separator, air separator, and dry mechanical cleaning. The flakes then undergo a cold washing step including a float-sink-separation and are collected before entering regranulation. The flakes were dried at 80 °C for 6 h and cut to a target size of 10–20 mm using an MDSi 410/200 cutting mill from Hellweg Maschinenbau GmbH & Co. KG, Roetgen, Germany. NaOH was supplied by Carl Roth GmbH + Co. KG, Karlsruhe, Germany. CTAC was supplied by Thermo Fisher Scientific, Langerwehe, Germany. Cirkit Wash 01 was kindly provided by Siegwerk Druckfarben AG & Co. KGaA, Siegburg, Germany. TEGO CYCLE WA111 and TEGOMER DA850 were kindly provided by Evonik Industries AG, Essen, Germany.

### 2.2. Deinking Trials

The washing trials are based on the DIN SPEC 91496 but modified to account for the peculiarities of PCW flakes [29,30]. The water temperature and washing times varied in three steps (30 °C, 60 °C, 80 °C and 15 min, 60 min, 120 min, respectively). A single transparent virgin PE flake (provided by Siegwerk Druckfarben AG & Co. KGaA, Siegburg, Germany) was added to each washing trial to detect recolouring of the flakes due to dissolved inks. Due to the relatively low concentrations of printing inks in PCW flake fractions compared to printed model films, no trial showed any signs of recolouring.

NaOH was used at concentrations of 1% and 2%. The surfactants were kept at a fixed concentration above the critical micelle concentration (cmc) [5,27], which was proposed by the DIN SPEC 91496 to be 0.2 wt.% in the case of CTAC [30] or by the respective surfactant suppliers. Cirkit Wash 01 (hereafter referred to as CW01) was used at a concentration of 0.25%. Non-ionic surfactants TEGO CYCLE WA111 (hereafter referred to as WA111) and TEGOMER DA850 (hereafter referred to as DA850) were used at concentrations of 0.5% and 0.25%, respectively. The deinking parameters and surfactants are summarised in Table 1.

In a 2 L beaker, the respective surfactant was dissolved in a solution (1 L) of NaOH, and the mixture was held at the target temperature. A total of 10 g of PCW flakes were stirred in the solution at 200 min^−1^ for the target washing time. The flakes were then removed from the washing solution, rinsed with 0.5 L of water, and dried in a convection oven from Thermo Fisher Scientific, Langerwehe, Germany, at 80 °C for 6 h.

### 2.3. Evaluation of Ink Removal

Transmission measurements are conducted on a Perfection V750 Pro from SEIKO Epson Corporation, Suwa, Japan. Two-gram batches of the flakes are placed on the scanning surface and manually distributed to ensure an even distribution of flakes with minimal overlapping. This measurement is repeated with the next 2 g batch of flakes, yielding a total of 4 scans and 8 g of scanned flakes per experiment. Greyscale images of the scans are created, and all pixels below a grey value threshold of 240 are evaluated. This ensures that only flake pixels and no background are evaluated [29]. Using grey values from all scans of a respective experiment, normalised distributions are extracted that can be quantitatively described via statistical characteristics.

### 2.4. Statistical Analysis

A previous study has already demonstrated that the median grey value Q2 allows for a quantitative comparison of deinking experiments [29]. A multiple linear regression is set up with Q2 as the target value using JMP Student Edition 19 (SAS Institute, Cary, NC, USA). The investigated parameters (lye concentration, surfactant, washing time, and water temperature) from the full factorial design are considered. Main effects, corresponding to the respective parameters, and interactions between two or more parameters can be considered in the regression model. The model quality is evaluated using the coefficient of determination *R*^2^. It indicates the proportion of the total variance in the median grey values that is explained by the respective regression model. High values of *R*^2^ indicate a well-fitted model, whereas lower values suggest a higher proportion of unexplained variance. The inclusion of third-order interactions increases *R*^2^ but may lead to overfitting due to a loss of degrees of freedom in the linear regression model. The adjusted coefficient of determination *R*^2^*adj* accounts for the loss of degrees of freedom and must thus be considered to investigate whether the inclusion of third-order interactions proves beneficial for the description of the deinking effects [31]. Furthermore, the root mean squared error *RMSE* is used to indicate the mean deviation of the model values from the real values. Parameters are considered significant when the *p*-value is below 0.05. The logworth, which is defined as −log_10_(p), serves as transformation to visualise *p* values in a pareto diagram. Consequently, a logworth above log_10_(0.05) = 1.30 indicates that the respective parameter is significant. Relevant effects are depicted in main effect and interaction diagrams with the least squares means (LSM) and the standard error that quantifies the prediction uncertainty of the calculated mean values [32,33,34].

## 3. Results and Discussion

The investigated effect terms and statistical parameters for the multiple linear regression model are listed in Table 2.

**Figure 2:**
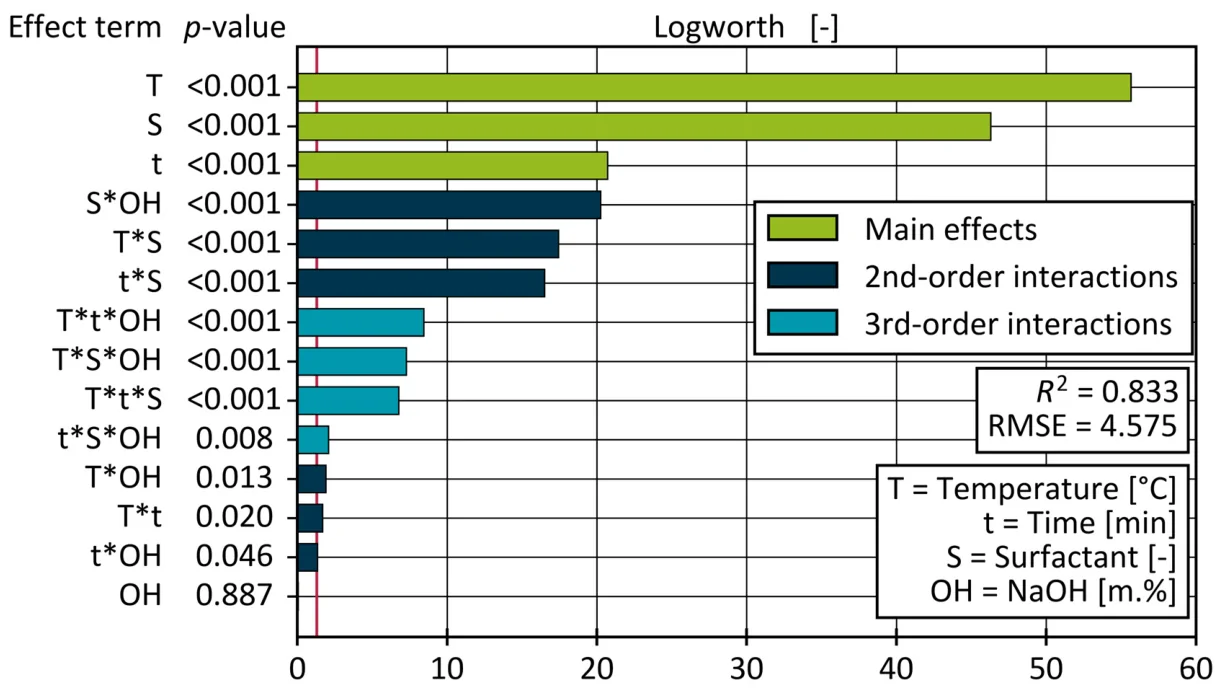
Effect terms with their respective *p*-value and logworth that were included in the model of the statistical analysis. Parameters are considered significant when the *p*-value is below 0.05 and the logworth is above 1.30.

The inclusion of third-order interactions leads to a decrease in *RMSE* of 0.9464 and an increase in *R*^2^ of 0.1031, indicating a better fitting model. More importantly, *R*^2^*adj* shows an increase of 0.0935, which indicates that the model extension does not lead to overfitting. It can be concluded that the inclusion of third-order interactions proves beneficial to describe the deinking effects [31]. Consequently, the statistical analysis was set up to include all relevant main effects and second-order as well as third-order interactions. Effect terms and their logworth are depicted as a pareto diagram in Figure 2.

**Figure 3:**
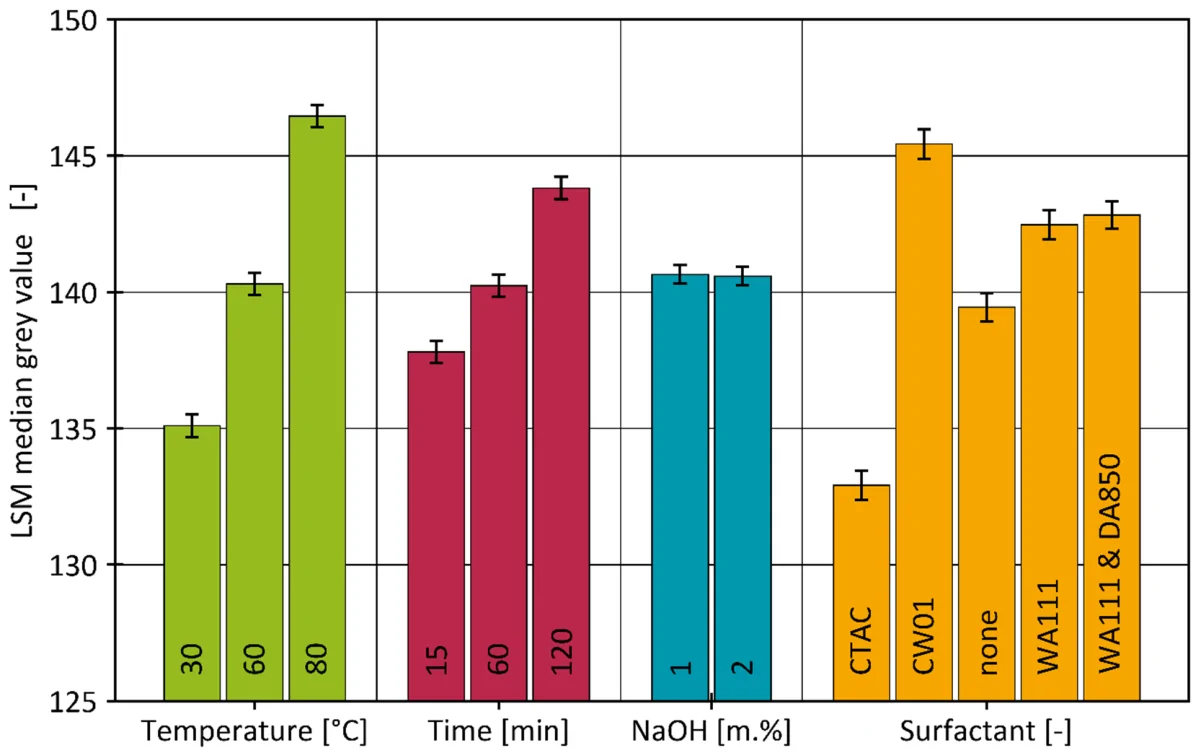
Least squares means of the median grey values (LSM Q2) and their respective standard errors for all main effects. The median grey value of PCW flakes prior to deinking was calculated to be 125 ± 2.59.

The transformation of *p*-values to the logworth allows visualisation of the significance of the individual effect terms. As depicted in Figure 2, all main effects and interactions except for the concentration of NaOH are significant and thus included into the regression model. Since the interactions with the lye concentration are significant, the main effect is also kept in the model. LSM values with their respective standard errors are depicted in Figure 3.

LSM median grey values (LSM Q2) depicted in Figure 3 are calculated from the means of the median grey values for all experiments at the respective factor level and therefore provide an overview of the relevant effects. Opposing interactions, such as those that may occur between a surfactant and the concentration of NaOH, can only be accounted for in interaction diagrams. The influence of the lye concentration is therefore discussed below in Figure 6. Increases in both temperature and washing time yield higher LSM Q2. The positive effect of increased temperatures on deinking efficiency has been demonstrated in various publications [24,35,36]. Furthermore, it was shown that the deinking of surface-printed films proceeds relatively quickly. Dissolved inks can reversibly interact with the flakes, causing staining and thus a recolouring of the flakes. Especially for experiments using uniformly printed model films, the high concentration of dissolved inks in the washing solution enables staining of the flakes with increased washing times and shifts the equilibrium towards stained flakes in comparison to PCW flakes [5,29,36,37].

**Figure 4:**
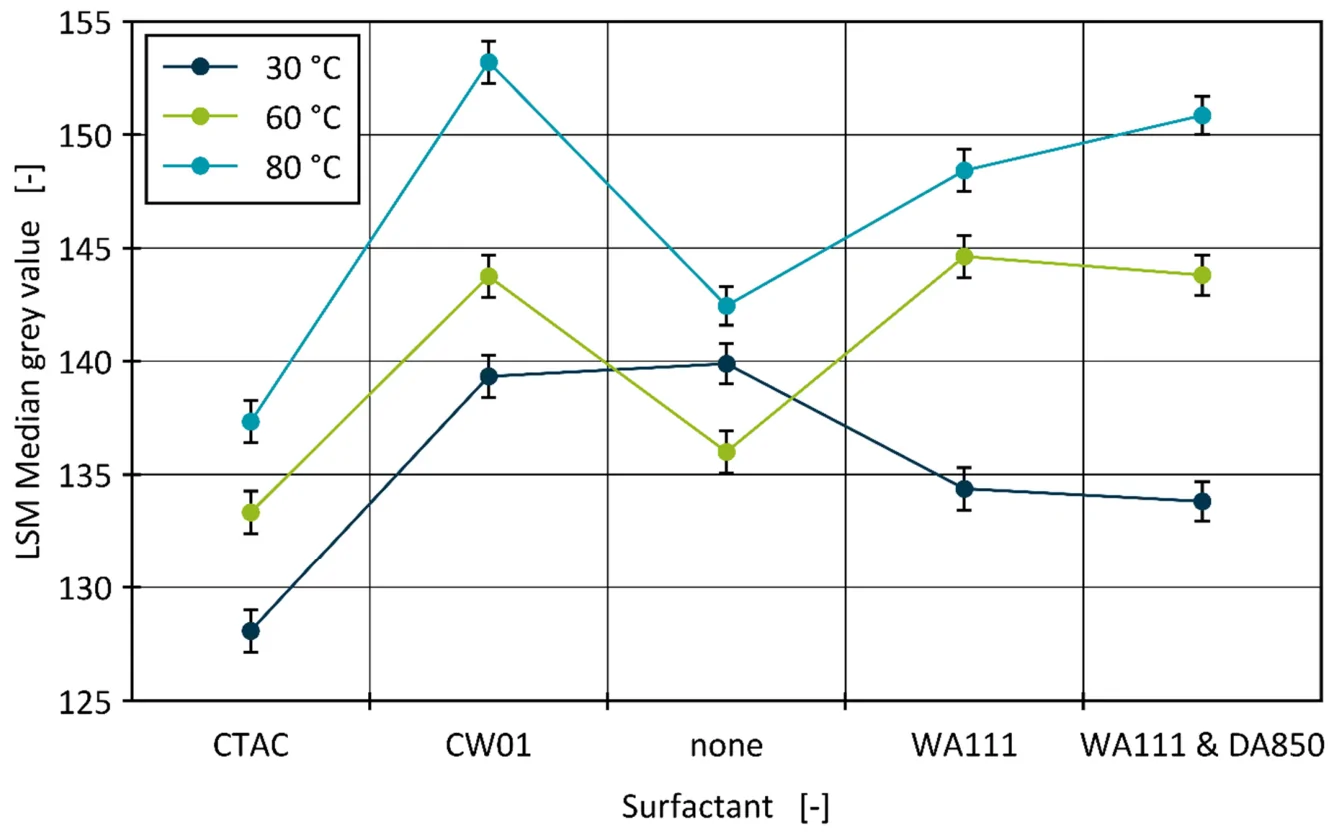
Least squares means of the median grey values and their respective standard errors for the interactions of the surfactants and the water temperatures.

Most deinking publications demonstrate the best deinking efficiencies for cationic surfactants. Quaternary ammonium-based surfactants like CTAC and its derivatives show consistently high deinking efficiencies, with CTAC being proposed in the DIN SPEC 91496 [5,24,30,35,36,38]. Among the parameters examined, CTAC shows the lowest deinking efficiency, with median grey values below the experiments that do not include any surfactant. This discrepancy indicates that the deinking of PCW flakes introduces additional factors that influence the ink removal compared to model films. Possibly, some waste residues on PCW flakes (fats, oils, etc.) have strong interactions with CTAC. If the formation of micelles with contaminants is energetically favoured compared to the adhesion and stabilisation of the binding agent, surfactant molecules would be partially deactivated and cannot engage in the deinking mechanism. CW01 is a surfactant formulation based on CTAC, the zwitterionic lauryldimethylamine oxide (LDAO), which is known to be highly hydrophilic, and Poly(oxy-1,2-ethanediyl), α-(2-propylheptyl)-ω-hydroxy-, which is a non-ionic ethoxylated alcohol that is industrially used as a cleaning agent and degreaser [39,40,41,42]. It can be hypothesised that the high deinking efficiency of CW01 is attributed to the combination of surfactants with different affinities that prove more robust to the various contaminants present in PCW flakes. The non-ionic surfactant formulations WA111 and WA111 and DA850 show similar mean deinking efficiencies that are between those of CW01 and experiments with no surfactant added. The lower LSM Q2 are partially caused by the low deinking efficiencies of the non-ionic surfactants at lower temperatures, which are outlined in Figure 4.

LSM Q2 depicted in Figure 4 widely confirm the trend discussed above that higher water temperatures lead to better deinking results, except for the points of 30 °C and 60 °C with no surfactant added. This reversed effect could be explained by the change in the deinking mechanism that results from the absence of surfactants. With only NaOH present in the washing solution, the hydrolysis of the binding agent’s covalent bonds is expected to be the dominating process for ink removal. This effect alone should lead to the same trend, with higher hydrolysis rates and, more importantly, higher diffusion rates of NaOH into the NC matrix with higher temperatures [24,43,44,45]. However, it has been postulated in literature that the glass transition or a similar phase transition of NC-based binding agents takes place at temperatures around 50 °C [46,47,48]. A softening of the binding agent could lead to an increased adhesion on the flake surface or a slower diffusion of the washing solution that hinders its removal from the flake within this temperature range but is compensated for by the higher hydrolysis rate at 80 °C. The addition of a surfactant reduces the interfacial energy between flake and binding agent, which might explain why this anomaly can only be observed when no surfactants are added.

**Figure 5:**
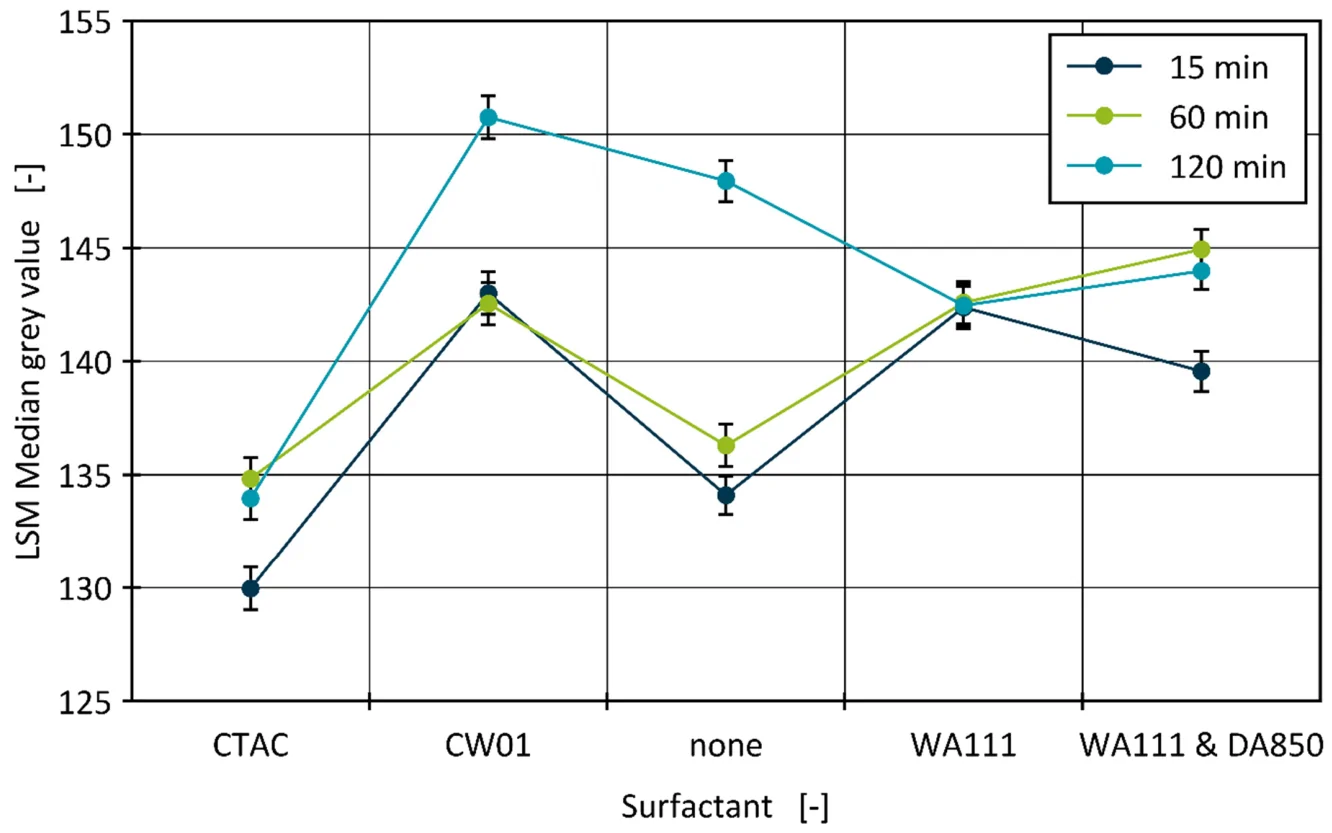
Least squares means of the median grey values and their respective standard errors for the interactions of the surfactants and the washing times.

LSM Q2 of the non-ionic surfactant formulations at 30 °C are lower than the experiments with no surfactant added. This can be explained by a limited solubility of the polymer-based surfactant at that temperature, which was observable in the experiments in the form of a cloudy washing solution. This hypothesis is supported by the sharp increase of the LSM Q2 at 60 °C. The interactions of the washing time and surfactants are depicted in Figure 5.

Within the parameters investigated, longer washing times generally lead to higher or equal LSM Q2. Without staining taking place in the deinking process, a stable equilibrium should establish itself in which surfactant molecules are present in micelles, stabilising printing inks as well as waste residues, and adsorbed onto interfaces. The time required to reach the equilibrium state is determined by the rate of ink removal processes, while the position of the equilibrium is determined by the surfactant’s ability to stabilise the dissolved inks. Consequently, significant increases in the LSM Q2 with increased washing time indicate that the deinking equilibrium is not yet established.

**Figure 6:**
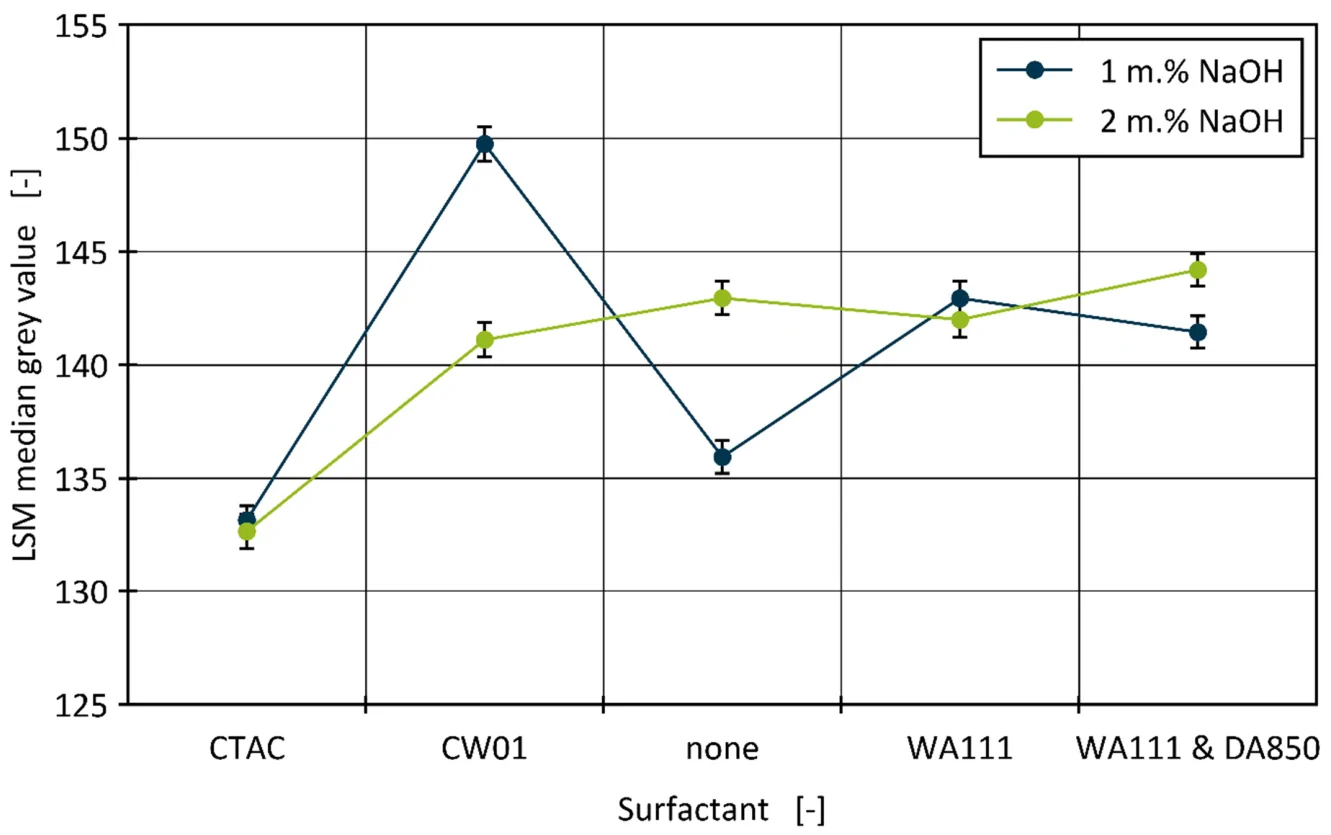
Least squares means of the median grey values and their respective standard errors for the interactions of the surfactants and the concentrations of sodium hydroxide.

The non-ionic surfactant WA111 shows no change in deinking efficiency for increased washing times, indicating quick ink removal. With the addition of DA850, LSM Q2 are lower at 15 min but higher at 60 min and 120 min. Since DA850 is used to stabilise dissolved printing inks, it can be hypothesised that a greater amount of printing ink can be contained in micelles, and the equilibrium is shifted to allow for greater ink removal. However, with multiple surfactants present, it is possible that the deinking rate decreases due to surfactant-surfactant interactions or steric hindrance at the printing ink interfaces. For CTAC, it is shown that LSM Q2 increase after 15 min but stay mostly unaffected after 60 min. The experiments with NaOH only show a slight increase after 60 min and a sharp increase after 120 min. With no surfactant present to decrease the interfacial energy, the diffusion of NaOH into the binding agent and the removal of the printing ink layer from the flake surface should proceed at a lower rate. The diffusion is the rate-determining step, and the hydrolysis mechanism is necessary for the establishment of the equilibrium. Therefore, it can be concluded that the deinking rate decreases significantly when no surfactant is present [43,44,45]. A similar trend is observed for the experiments using CW01, although the same explanation cannot apply here. The LSM Q2 after 15 and 60 min are roughly equal, but a sharp increase is shown after 120 min. While it is possible that the hydrolysis of the binding agent allows for increased amounts of stabilised molecules in micelles, this effect should lead to an increase in the LSM Q2 also after 60 min. In the case of CW01, it is hypothesised that the trend shown in Figure 5 is overshadowed by the averaging of experiments conducted at different lye concentrations. This is due to the strong dependency of CW01 on the lye concentration, which is discussed in the following. The interactions of lye concentrations and surfactants are shown in Figure 6.

**Figure 7:**
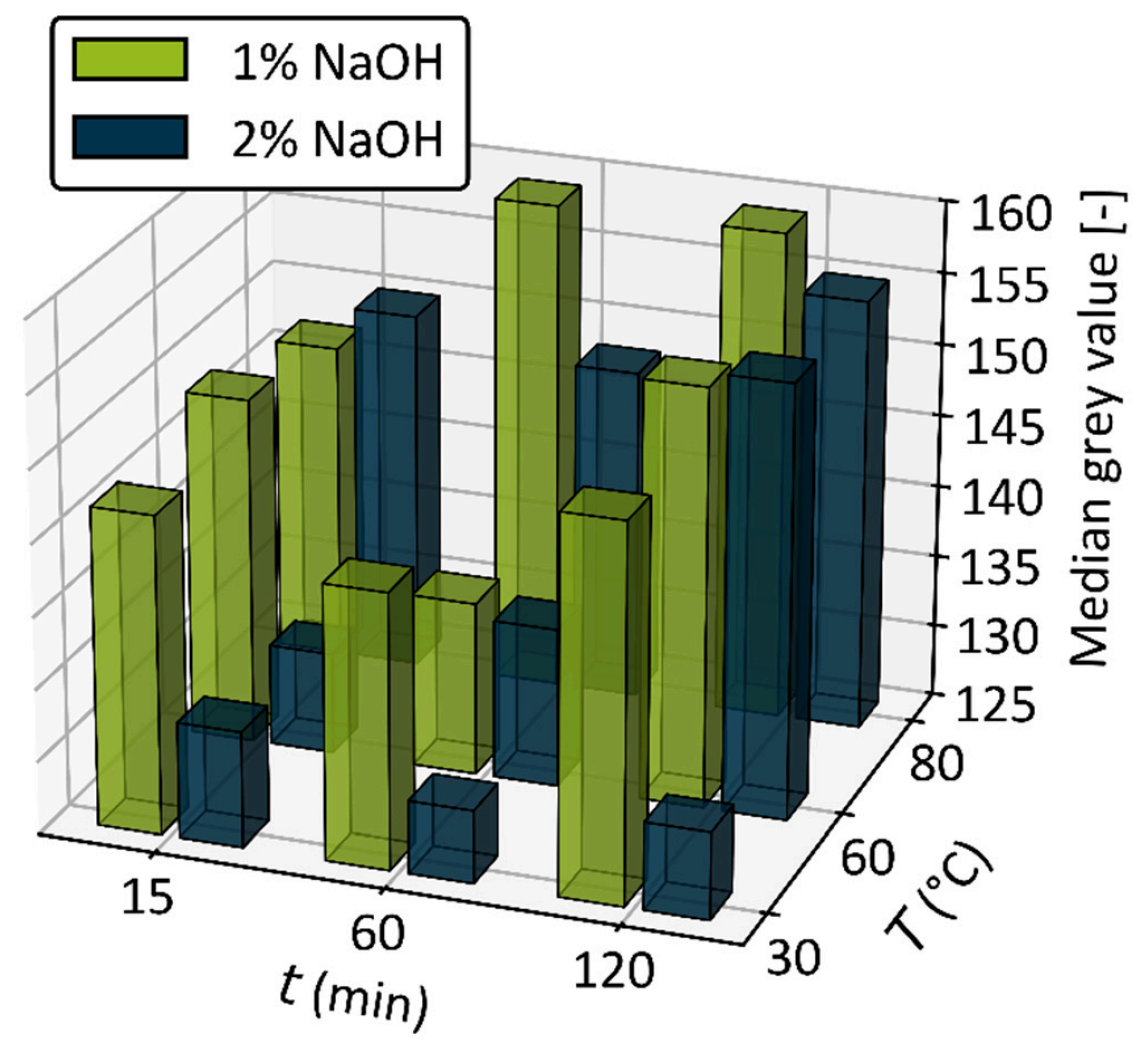
Median grey values for the interactions of the washing time, temperature, and the concentrations of sodium hydroxide for experiments using the surfactant CW01.

An increased concentration of NaOH leads to increased LSM Q2 for experiments with no surfactant added. This is in accordance with the hypothesis of the hydrolysis of the binding agent and more precisely the diffusion of the hydroxy ions into the binding agent matrix being the rate-determining step when no surfactant is present [43,44,45]. With the addition of non-ionic surfactants, the LSM Q2 at 1% NaOH increase significantly, while the LSM Q2 at 2% NaOH are in a similar range to those with no surfactant added. Since these LSM Q2 are averaged for all washing times, these results show the positive effect of decreasing the interfacial energy on ink removal, especially at lower lye concentrations. For cationic surfactants, a different effect can be observed. The increase in lye concentration has no effect on the LSM Q2 in the experiments using CTAC and causes a decrease in the LSM Q2 in the experiments using CW01. This effect contrasts with the past experiments on model films in which higher concentrations of NaOH increase the deinking efficiency for cationic surfactants as well [24,30,36]. Since the investigated PCW flakes contain an overall lower amount of printing inks compared to the model films used in literature, it can be hypothesised that there is an excess of hydroxy ions in the experiments using 2% NaOH. When the maximum amount of hydroxy ions interacting with the printing ink is reached, residual ions are likely to interact with the cationic surfactant. Since the cetrimonium ion cannot be deprotonated, the interaction could lead to an increase of micelle stability and thus increase the activation energy for the deposition of the cationic surfactant on the flake-ink interface. To investigate this hypothesis, the interactions of the washing time, water temperature, and lye concentration are depicted for the experiments in Figure 7.

**Figure 8:**
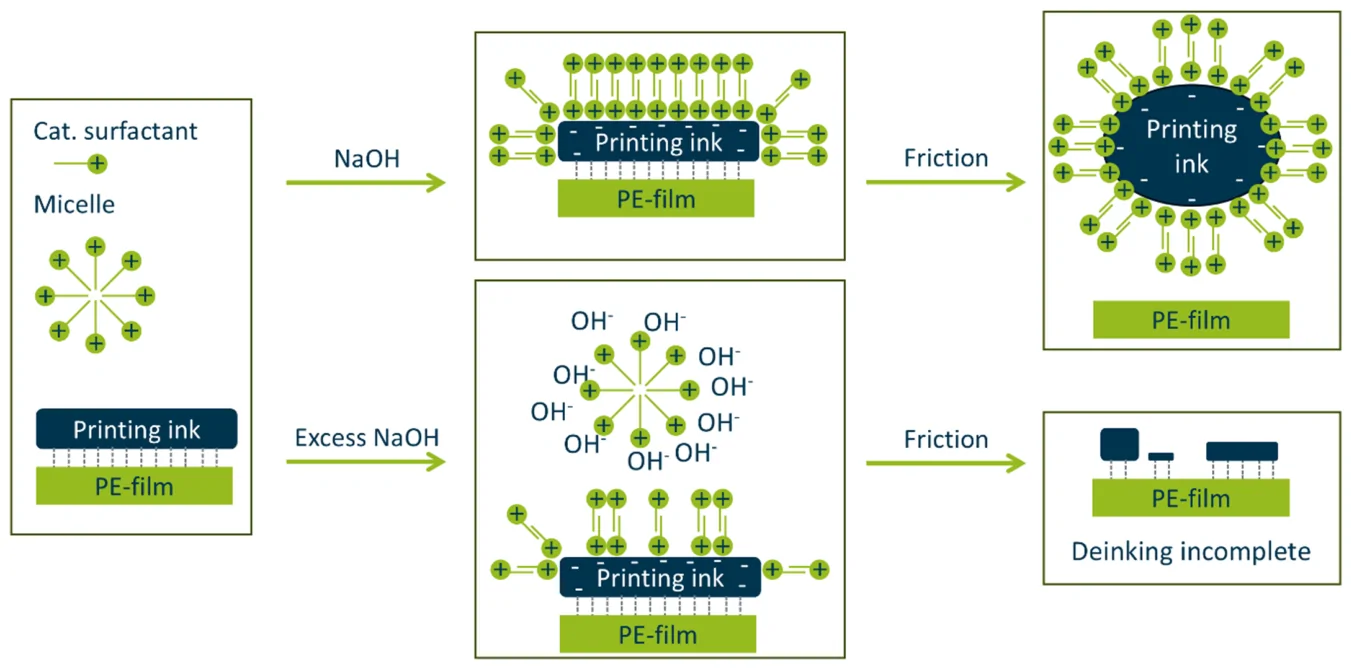
Deinking mechanism for physically drying inks, drawn after [24] expanded with the proposed effect of excess NaOH on the deinking using cationic surfactants. NaOH interacts with the binding agent in the ink layer, creating negative charges on the ink layer surface. Excess hydroxy ions might stabilise the micelles of cationic surfactants and decrease the deposition of cationic surfactant molecules on the flake-ink interface. The surfactant deactivation might result in a lower rate of ink removal with the hydrolysis of the binding agent becoming more dominant in the deinking process.

The comparison of the median grey values at different lye concentrations shows a stronger dependence on the washing time and water temperature for experiments at 2% NaOH. Especially at 30 °C, low median grey values between 130 and 133 are achieved. At 60 °C, higher grey values are achieved only at a washing time of 120 min, yielding 155. When compared with the dependencies discussed for the experiments using NaOH only, these results indicate that the hydrolysis of the printing ink is the dominating mechanism at 2% NaOH. This supports the hypothesis that at higher lye concentrations, cetrimonium ions are partially deactivated for deinking due to strong interactions with hydroxy ions. In Figure 8, the deinking mechanism is expanded to visualise this proposed effect.

## 4. Conclusions

The systematic investigation of the deinking of household PCW flakes at varying parameters (water temperature, washing time, and lye concentration) and using industrially available surfactants offers insights into the deinking mechanism on PCW flakes, which are valuable for adapting deinking processes on an industrial scale. The multiple linear regression model with an *R*^2^ of 0.833 proved the significance of deinking parameters. In conjunction with the literature investigating the deinking on uniformly printed model films, higher water temperatures yield higher LSM Q2 (i.e., higher deinking efficiencies). Due to the overall lower amount of printing ink in PCW flakes compared to model films, no staining was observed. Consequently, higher washing times lead to higher LSM Q2 in the conducted experiments. Interactions between the respective surfactants and the deinking parameters provided insights into the deinking mechanism. In contrast with experiments on model films, the addition of CTAC showed overall poor deinking. This indicates that without additional surfactants added, cetrimonium ions might be deactivated due to strong interactions with residual contaminants present on the PCW flakes. In the range of the experiments conducted, the non-ionic surfactants showed overall good deinking results with a strong dependence on the water temperature only. This is most likely due to the limited solubility of the polymer-based surfactant molecules at lower temperatures. Experiments using the surfactant formulation CW01 showed the highest deinking efficiency achieved in this study, with a median grey value of 159 at 1% NaOH and 80 °C, reached after both 60 min and 120 min of washing. While experiments conducted with 1% NaOH consistently yielded high median grey values, a strong dependence on the water temperature and washing time was observed for the experiments at 2% NaOH. It is indicated that this effect is attributable to the lower amount of printing inks in PCW flakes compared to model films. The addition of lye concentrations above 1%, which are common in the literature, causes an excess when deinking PCW flakes. Consequently, cationic cetrimonium surfactants might interact strongly with dissolved hydroxy ions, therefore limiting their availability for deinking.

Building on these results, the necessity to adapt the lye concentration of deinking processes to the amount of printing ink in the given material flow becomes apparent. An optimisation of the lye concentration allows for benefiting from the hydrolysis of the binding agent molecules without sacrificing the reduction of the interfacial energy, introduced by the surfactant. However, since PCW is highly heterogeneous in its composition and, most importantly, its amount of residues and printing ink, a continuous optimisation of deinking parameters is not feasible on an industrial scale. Based on the results discussed in this study, a deinking process should adapt the lye concentration to the estimated amount of printing inks present in the PCW material flow, combined with a surfactant formulation that proves robust in the range of deinking parameters used. Investigating additional household PCW streams, such as flexible PE from other sources or flexible PP waste, would be a valuable next step to test the hypotheses made in this study. This would also increase the transferability to industrial processes by deriving more generally applicable guidelines for adapting deinking parameters.

In particular, the hypothesised deactivation of cationic surfactants through interactions with waste residues suggests that the deinking behaviour observed here could differ for material streams with a different type or amount of contamination.

Investigating additional household PCW streams, such as flexible polypropylene (PP) waste, would therefore be a valuable next step, both to assess the transferability of the identified deinking mechanisms and to derive more generally applicable guidelines for adapting and optimising industrial deinking processes across different PCW material streams.

## Figures and Tables

**Table 1:** Overview of deinking parameters and surfactants, investigated in a full-factorial design, yielding a total of 90 experiments.

Temperature	Washing Time	NaOH Concentration	Surfactant	Surfactant Concentration	Surfactant Type
30 °C	15 min	1 wt.%	CTAC	0.2 wt.%	Cationic
60 °C	60 min		CW01	0.25 wt.%	Cationic, zwitterionic, non-ionic
		2 wt.%	none	-	-
80 °C	120 min		WA111	0.5 wt.%	Non-ionic
			WA111 & DA850	0.5 wt.% & 0.25 wt.%	Non-ionic

**Table 2:** Main effects and second-order and third-order interactions as well as coefficients of determination and root mean squared errors for multiple linear regression models, including main effects and either second-order interactions or second- and third-order interactions.

	Main Effects	2nd-Order Interactions	3rd-Order Interactions
Effect terms	Temperature (T) [°C] Time (t) [min] Surfactant (S) [−] NaOH (OH) [m.%]	S*OH T*S t*S T*OH T*t t*OH	T*t*OH T*S*OH T*t*S t*S*OH
*R* ^2^	-	0.7299	0.8333
*R* ^2^ *adj*	-	0.7019	0.7952
*RMSE*	-	5.5214	4.575

## Data Availability

The original contributions presented in this study are included in the article. Further inquiries can be directed to the corresponding author.

## Abbreviations

The following abbreviations are used in this manuscript:

CIELab	International Commission on Illumination Lab
cmc	critical micelle concentration
CTAC	cetrimonium chloride
CW01	Cirkit Wash 01
DA850	TEGOMER DA850
EVOH	ethylene vinyl alcohol
LDAO	lauryldimethylamine oxide
LSM	least squares mean
LSM Q2	least squares mean median grey value(s)
NaOH	sodium hydroxide
NC	nitrocellulose
NIAS	non-intentionally added substances
PCR	post-consumer recyclate
PCW	post-consumer waste
PE	polyethylene
PET	polyethylene terephthalate
PP	polypropylene
PPWR	packaging and packaging waste regulation
*R*^2^	coefficient of determination
*R*^2^*adj*	adjusted coefficient of determination
*RMSE*	root mean squared error
WA111	TEGO CYCLE WA111

## References

[1] 2023 Flexible Films Market in Europe: State of Play. PLastic Recyclers Europe, 2023. https://www.plasticsrecyclers.eu/wp-content/uploads/2023/09/2023-Flexible-Films-Market-in-Europe_State-of-Play_September-2023.pdf.

[2] Horodytska O., Valdés F.J., Fullana A. (2018). Plastic flexible films waste management—A state of art review. Waste Manag..

[3] Hahladakis J.N., Velis C.A., Weber R., Iacovidou E., Purnell P. (2018). An overview of chemical additives present in plastics: Migration, release, fate and environmental impact during their use, disposal and recycling. J. Hazard. Mater..

[4] Żołek-Tryznowska Z., Izdebska J., Thomas S. (2016). 4—Additives for Ink Manufacture. Printing on Polymers.

[5] Ügdüler S., Van Laere T., De Somer T., Gusev S., Van Geem K.M., Kulawig A., Leineweber R., Defoin M., Van den Bergen H., Bontinck D. (2023). Understanding the complexity of deinking plastic waste: An assessment of the efficiency of different treatments to remove ink resins from printed plastic film. J. Hazard. Mater..

[6] Stoffstrombild Kunststoffe in Deutschland 2023. Conversio, 2024. https://www.bkv-gmbh.de/files/bkv/studien/Kurzfassung%20Stoffstrombild%202023.pdf.

[7] Dahlmann R., Hopmannn C. (2025). Nachhaltige Kunststoffverpackungen aus Post Consumer-Rezyklaten.

[8] Berg E. (2023). Material analysis of polyethylene recyclates for use in film extrusion. ExxonMobil European Research & Development Days.

[9] Berg E., Leuchtenberger L., Dahlmann R. Influence of varying material components in representative polyethylene recyclates on the mechanical properties of blown films. Proceedings of the 32nd International Colloquium Plastics Technology.

[10] Dahlmann R., Berg E., Schön M. Usage potentials for different qualities of recyclates in plastics packaging. Proceedings of the 31st International Colloquium Plastics Technology.

[11] Cabanes A., Valdés F.J., Fullana A. (2020). A review on VOCs from recycled plastics. Sustain. Mater. Technol..

[12] Designing for a Circular Economy. CEFLEX, 2025. https://guidelines.ceflex.eu/assets/public_docs/D4ACE_Guidelines_Executive%20Summary_Sep25.pdf.

[13] Handbook on Decorative Technologies. RecyClass, 2023. https://recyclass.eu/wp-content/uploads/2025/10/Handbook-on-decorative-technologies-for-PEPP-flexibles-v1.0.pdf.

[14] Regulation (EU) 2025/40 of the European Parliament and of the Council of 19 December 2024 on Packaging and Packaging Waste. https://eur-lex.europa.eu/legal-content/EN/TXT/?uri=OJ:L_202500040&pk_campaign=todays_OJ&pk_source=EUR-Lex&pk_medium=X&pk_co%E2%80%A6.

[15] Printing Inks and Plastic Recycling—Q & A. EUPIA, European Printing Ink Association, 2021. https://www.eupia.org/wp-content/uploads/2022/09/Printing_inks_and_Plastic_Recycling_-_Q___A.pdf.

[16] Rempel E., Marm A. Fit for Recycling—How Inks Enable a Circular Economy. Drupa Cube, 2024. https://www.wirsindfarbe.de/fileadmin/user_upload/Fit_for_recycling_-_How_inks_enable_a_Circular_Economy.pdf.

[17] Guo J., Luo C., Chong Z.K., Alassali A., Kuchta K. (2024). Thermal degradation and hydrolysis depolymerization of printing ink components for plastic packaging in recycling processes: A review. Front. Environ. Sci. Eng..

[18] Cecon V.S., Da Silva P.F., Curtzwiler G.W., Vorst K.L. (2021). The challenges in recycling post-consumer polyolefins for food contact applications: A review. Resour. Conserv. Recycl..

[19] Berg E., Leuchtenberger L., Dahlmann R. Investigation of gel formation and degradation of representative polyolefin recyclates. Proceedings of the ANTEC^®^ 2024.

[20] Lisiecki M., Belé T.G.A., Ügdüler S., Fiorio R., Astrup T.F., De Meester S., Ragaert K. (2024). Mechanical recycling of printed flexible plastic packaging: The role of binders and pigments. J. Hazard. Mater..

[21] Strangl M., Ortner E., Buettner A. (2019). Evaluation of the efficiency of odor removal from recycled HDPE using a modified recycling process. Resour. Conserv. Recycl..

[22] Roosen M., De Somer T., Demets R., Ügdüler S., Meesseman V., Van Gorp B., Ragaert K., Van Geem K.M., Walgraeve C., Dumoulin A. (2021). Towards a better understanding of odor removal from post-consumer plastic film waste: A kinetic study on deodorization efficiencies with different washing media. Waste Manag..

[23] Roosen M., Harinck L., Ügdüler S., De Somer T., Hucks A.-G., Belé T.G.A., Buettner A., Ragaert K., Van Geem K.M., Dumoulin A. (2022). Deodorization of post-consumer plastic waste fractions: A comparison of different washing media. Sci. Total Environ..

[24] Guo J., Luo C., Wittkowski C., Fehr I., Chong Z., Kitzberger M., Alassali A., Zhao X., Leineweber R., Feng Y. (2023). Screening the Impact of Surfactants and Reaction Conditions on the De-Inkability of Different Printing Ink Systems for Plastic Packaging. Polymers.

[25] Gecol H., Scamehorn J.F., Christian S.D., Grady B.P., Riddell F.E. (2004). Use of surfactants to remove water-based inks from plastic film: Effect of calcium ion concentration and length of surfactant hydrophobe. Colloid Polym. Sci..

[26] Strangl M., Ortner E., Fell T., Ginzinger T., Buettner A. (2020). Odor characterization along the recycling process of post-consumer plastic film fractions. J. Clean. Prod..

[27] Chotipong A., Scamehorn J.F., Rirksomboon T., Chavadej S., Supaphol P. (2007). Removal of solvent-based ink from printed surface of high-density polyethylene bottles by alkyltrimethylammonium bromides: Effects of pH, temperature, and salinity. Colloids Surf. A Physicochem. Eng. Asp..

[28] Izdebska J., Izdebska J., Thomas S. (2016). 1—Printing on Polymers: Theory and Practice. Printing on Polymers.

[29] Zimmer S., Seifert L., Dahlmann R. (2026). Deinking of Post-Consumer Waste Flakes—Objective Assessment of Ink Removal on Inhomogeneous Film Fractions. Polymers.

[30] (2024). Recycling of Printed Polymer Packaging—Evaluation of Deinking Using a Test Procedure.

[31] Greene W.H. (2002). Econometric Analysis.

[32] Shardt Y.A.W., Weiß H. (2021). Versuchsplanung. Methoden der Statistik und Prozessanalyse.

[33] Janczyk M., Pfister R. (2020). Inferenzstatistik Verstehen.

[34] Willmott C.J., Matsuura K. (2006). On the use of dimensioned measures of error to evaluate the performance of spatial interpolators. Int. J. Geogr. Inf. Sci..

[35] Syed Ali S.A., Ilankoon I.M.S.K., Zhang L., Tan J. (2025). Response surface methodology optimisation of catanionic surfactant mixtures in de-inking packaging plastic waste. J. Hazard. Mater..

[36] Guo J., Kim Y., Chong Z.K., Alassali A., Chacon J.P., Gottschalk D., Kitzberger M., Kuchta K. (2022). Quality Changes of Low-Density Polyethylene (LDPE) Recyclates from the Pretreatment Process with a Cationic Surfactant and a Nonionic Surfactant as Cleaning Agents Upstream of Extrusion. Processes.

[37] De Somer T., Ügdüler S., Manhaeghe D., Harinck L., Van Geem K.M., Kulawig A., Leineweber R., De Meester S. (2026). Controlling colorant staining during the de-inking of flexible plastic packaging. Sep. Purif. Technol..

[38] Syed Ali S.A., Ilankoon I.M.S.K., Zhang L., Tan J. (2024). Understanding de-inking in packaging plastic recycling: Bridging the gap in resource conservation and establishing average hazard quotient. J. Hazard. Mater..

[39] Birnie C.R., Malamud D., Schnaare R.L. (2000). Antimicrobial Evaluation of N-Alkyl Betaines and N-Alkyl-N,N-Dimethylamine Oxides with Variations in Chain Length. Antimicrob. Agents Chemother..

[40] Hoffmann H., Findenegg G.H. (1990). Correlation between surface and interfacial tensions with micellar structures and properties of surfactant solutions. Interfaces in Condensed Systems.

[41] Kocherbitov V., Söderman O. (2006). Hydration of Dimethyldodecylamine-N-oxide:  Enthalpy and Entropy Driven Processes. J. Phys. Chem. B.

[42] Kocherbitov V., Veryazov V., Söderman O. (2007). Hydration of trimethylamine-N-oxide and of dimethyldodecylamine-N-oxide: An ab initio study. J. Mol. Struct. THEOCHEM.

[43] Christodoulatos C., Su T.-L., Koutsospyros A. (2001). Kinetics of the Alkaline Hydrolysis of Nitrocellulose. Water Environ. Res..

[44] Su T.-L., Christodoulatos C. (1996). Destruction of Nitrocellulose Using Alkaline Hydrolysis. Proceedings of the Enhancing Readiness Through Environmental Quality Technology, Hershey, PA, USA, 20–22 May 1996.

[45] Li J., Yin X., Liu Z., Gu Z., Niu J. (2019). Reaction yield model of nitrocellulose alkaline hydrolysis. J. Hazard. Mater..

[46] Tomaszewski W., Gołofit T., Ostrowski M. (2022). Thermochemistry of the binary system nitrocellulose + dimethyl 4,4′-dinitroheptanedioate. Thermochim. Acta.

[47] de Paula A.C., Uliana F., da Silva Filho E.A., Soares K., Luz P.P. (2019). Use of DMA-material pocket to determine the glass transition temperature of nitrocellulose blends in film form. Carbohydr. Polym..

[48] Zhanning J. (1992). The glass transition temperature measurement of nitrocellulose by torsional braid analysis. Propellants Explos. Pyrotech..

